# Impact of Novel Prebiotic Galacto-Oligosaccharides on Various Biomarkers of Colorectal Cancer in Wister Rats

**DOI:** 10.3390/ijms18091785

**Published:** 2017-08-31

**Authors:** Tahir Rasool Qamar, Sanaullah Iqbal, Fatima Syed, Muhammad Nasir, Habib Rehman, Muhammad Aamir Iqbal, Rui Hai Liu

**Affiliations:** 1 Department of Food Science and Human Nutrition, University of Veterinary & Animal Sciences, Punjab 54000, Pakistan; tahirnutritionist@gmail.com (T.R.Q.); fatimasyed46@gmail.com (F.S.); nasir@uvas.edu.pk (M.N.); aamir.iqbal@uvas.edu.pk (M.A.I.); 2 Department of Physiology, University of Veterinary & Animal Sciences, Punjab 54000, Pakistan; habibrehman@uvas.edu.pk; 3 Department of Food Science, Cornell University, Ithaca, NY 14850, USA; rl23@cornell.edu

**Keywords:** galacto-oligosaccharides, colorectal cancer, aberrant crypt foci, bacterial enzymes

## Abstract

Colorectal cancer (CRC) is one of the leading causes of cancer deaths around the globe. Bioactive food ingredients such as prebiotics have protective potential in colon cancer. Data on galacto-oligosaccharides (GalOS) against CRC are very limited and GalOS used in this study have β-1,6 and β-1,3 as major glycosidic linkages and, to our best knowledge, were never used before against any cancer treatment. This study aims to investigate the protective role of novel GalOS against various biomarkers of CRC including aberrant crypt foci (ACF), bacterial enzymes and short chain fatty acids (SCFA) in a rodent model induced with 1,2-dimethylhydrazine dihydrochloride (DMH). Inulin group was taken as positive control in present study to compare novel GalOS protective effects. GalOS doses of 76–151 mg and inulin doses of 114 mg were given to different groups treated with DMH. Results showed that ACF formation was significantly (*p* ≤ 0.05) less in high dose GalOS group (27.3%). GalOS also had protective effects against DMH-induced body weight loss and showed higher level of cecal and fecal SCFA (acetate, propionate and butyrate). High doses of GalOS also resulted in significant (*p* ≤ 0.05) reduction of bacterial enzymatic activities. Increased populations of beneficial bacteria (bifidobacteria and lactobacilli) and decreased concentrations of harmful bacteria were observed in all prebiotics treatment groups. It can be concluded that novel GalOS exhibit robust protective activity against ACF formation in vivo.

## 1. Introduction

Food plays important role in improving health status and to manage different diseases. In modern times, much importance has been given to investigate impact of food components on health. In recent years, functional foods have gained significant importance by scientists and consumers and are defined as “food ingredients that provide health benefits beyond traditional nutrients it contains” [1]. In many countries, herbal medicines are being combined with traditional foods to make functional foods and are also used in dietary supplements for various health benefits. Prebiotics are functional ingredients that are not digested by mammalian enzymes and reach in colon, fermented by selective microbes to improve growth of gastrointestinal microbiota for host well-being and health [2]. Prebiotics help in selective stimulation of beneficial bacteria especially lactobacilli and bifidobacteria as well as their saccharolytic metabolites such as short chain fatty acids (SCFA), particularly butyrate [3].

There are different types of prebiotics and are indigestible carbohydrates such as oligofructose, lactulose, isomalto-oligosaccharides, xylo-oligosaccharides, soybean oligosaccharides, inulin-type fructans, galacto-oligosaccharides (GalOS) and arabinoxylan-oligosaccharides [4]. Prebiotic GalOS, also called trans-galactosylated oligosaccharides (TOS), are composed of galactose and glucose molecules having a β-linkage and are produced by a process known as transgalactosylation of lactose catalyzed by β-galactosidase (β-gal) [5]. GalOS are principally composed of 2–8 saccharide units, with glucose being the terminal unit and other units are galactose [6].

Colorectal cancer (CRC) is a complex multistage disease and is considered a major cause of mortality in humans, irrespective of gender, among all cancer-related deaths around the globe [7,8]. There are several risk factors for colon cancer such as age, gender, physical activity, genetic factors, food habits, inflammatory bowel diseases and pro-carcinogens present in food supply chain and environment [9]. Diet has major influence on gut related cancers; therefore those foods which positively affect colonic health by modifying gut micro flora are considered useful strategy against CRC protection [10]. CRC has been induced chemically in various animal models successfully to study tumor development, risk factors of CRC and its prevention [11,12]. CRC development in both human and rodents is followed by a sequence of pathological changes, ranging from aberrant crypt foci (ACF), which are distinct microscopic lesions in mucosa, to malignant tumors [8,13]. ACF are paraneoplastic lesions found in colon mucosa of patients at high risk of CRC development and in animals treated with chemical carcinogens [14,15]. ACF can be induced chemically in rodent colon by single or multiple doses of 1,2-dimethylhydrazine dihydrochloride (DMH) or azoxymethane [8]. Detection and quantification of ACF has been proposed to identify chemo-preventive agents present in short as well as medium term bioassays of rodent colon carcinogenesis [11,14].

In the last few decades, food scientists and nutritionists have focused on prebiotics as natural dietary ingredients to improve smooth functioning of gastrointestinal tract and to protect colon cancer [16]. Most commonly investigated oligosaccharides for cancer protection are inulin and several in vivo studies have proven their protective effects against CRC [17,18,19]. However, very limited data are available for efficacy of GalOS against CRC. Furthermore, to the best of our knowledge, no study has been conducted so far using GalOS with β-1,6 and β-1,3 as major glycosidic linkages. Only two studies have been conducted by one research group using GalOS against CRC having β-1,4 as main linkage [20,21]. It is the reason that in present study, prebiotic inulin group was added as positive control to compare the protective effects of novel GalOS having β-1,6 and β-1,3 as main glycosidic linkage. This study aims to investigate protective effect of GalOS against various biomarkers of CRC including ACF, enzymatic activities of microbiota and short chain fatty acids (SCFAs) in a DMH induced rodent model. The main finding of our study is that GalOS have more protective effects against CRC at higher doses as compared to low dose.

## 2. Results

### 2.1. Body Weight Changes

**Figure 1:**
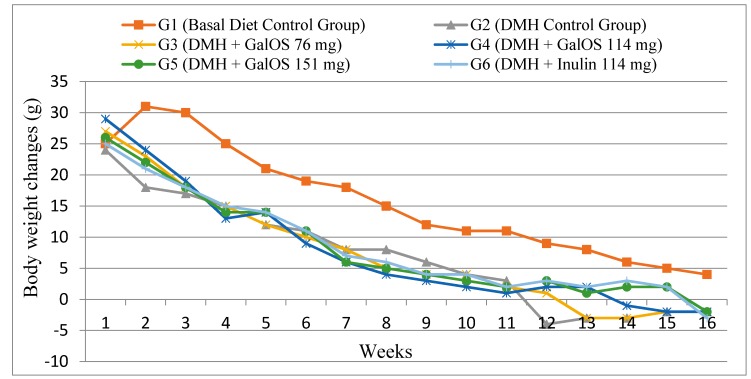
Body weight changes in different groups of animals throughout the experiment in 1,2-dimethylhydrazine dihydrochloride (DMH) treated and non-treated groups (*n* = 15).

Data of weekly body weight changes are shown in Figure 1. All groups were fed on basal diet ad libitum and no significant difference was observed in food intake among all groups. Group G1 (basal diet group) gained maximum body weight (*p* ≤ 0.05) as compared to all other groups. DMH showed high impact on lowering body weights of DMH treated animals and the negative control Group G2 (basal diet + DMH) gained the lowest body weight as compared to all other groups. Interestingly, those animals (Groups G3–G6) receiving prebiotics as treatments showed resistance against DMH effect on hindering body weight gain. Among GalOS groups, G5 group showed significant (*p* ≤ 0.05) increase in body weight gain as compared to Group G2. Group G6, which was receiving inulin as treatment, gained maximum body weight among all prebiotics groups and was significantly (*p* ≤ 0.05) higher as compared to Group G2, however this body weight gain was statistically similar to GalOS groups (G4 and G5).

### 2.2. Aberrant Crypt Foci (ACF)

**Figure 2:**
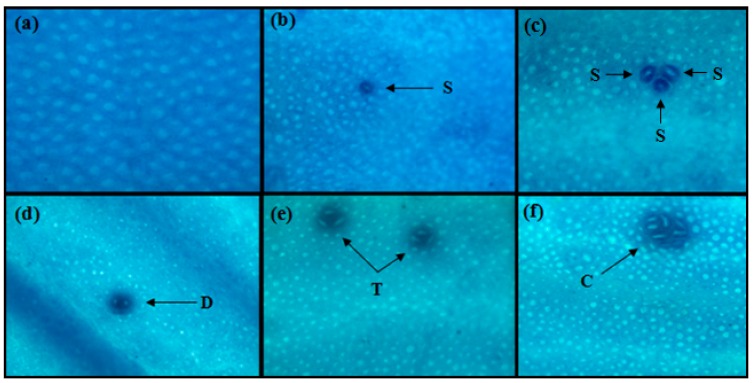
Microscopic view of the colons showing: (**a**) normal crypts of control animals; and (**b–f**) 1,2-dimethylhydrazine dihydrochloride (DMH) treated animals showing aberrant crypt foci stained with methylene blue (40×). Arrows indicate ACF: singlet (S), doublet (D), triplet (T), and cluster (C).

Microscopic examination of ACF in colon of DMH treated and non-treated animals is shown in Figure 2. Distribution of ACF in different parts of colon in all groups is summarized in Table 1. No ACF was detected in Group G1 (basal diet group). Maximum ACF in proximal (27.4 ± 2.10), middle (44.7 ± 2.98) and distal (119.1 ± 5.56) colon were observed in Group G2 (basal diet + DMH). Prebiotics treatment groups with high doses showed better results in hindering the occurrence of ACF in all parts of colon. Among GalOS treatment groups, G4 and G5 resulted in significant (*p* ≤ 0.05) reduction of ACF/colon as compared to Group G2. Group G6 receiving inulin as treatment was also significant (*p* ≤ 0.05) in reduction of ACF/colon as compared to Group G2. Maximum percentage of total ACF inhibition was shown by Group G6 (29.3%) and G5 (27.3%) as compared to G2 as well as other prebiotics groups (G3 and G4).

### 2.3. Short Chain Fatty Acids (SCFAs)

Results of SCFAs (acetate, butyrate and propionate) in cecal and fecal contents are given in Table 2. Those animals receiving prebiotics as treatment showed higher concentration of SCFAs, particularly high dose groups. Groups G5 and G6 showed significantly (*p* ≤ 0.05) higher concentration of acetate and butyrate in cecal contents as compared to Group G2 (basal diet + DMH). Only Group G6 had significantly (*p* ≤ 0.05) higher concentrations of propionate in cecum as compared to Group G2. In fecal contents, Groups G5 and G6 resulted in significant (*p* ≤ 0.05) higher concentrations of acetate and butyrate as compared to Group G2 While Groups G4–G6 were significantly (*p* ≤ 0.05) higher in concentrations of propionate in fecal contents as compared to Group G2.

### 2.4. Bacterial Enzyme

Table 3 summarizes bacterial enzyme activities in fresh cecal and fecal samples. Group G2 (basal diet + DMH) showed overall high bacterial enzymes activities in cecal and fecal contents. Among prebiotics treatment groups, Groups G5 and G6 showed lowest enzyme activities (for most enzymes) in both cecal and fecal contents. Groups G5 and G6 resulted in significant (*p* ≤ 0.05) lower contents of nitroreductase while only in Group G5 β-glucoronidase was significantly reduced in cecum as compared to Group G2. However, in the case of β-glucosidase, only Group G6 had significantly (*p* ≤ 0.05) lower contents in cecum compared to G2 group. In fecal samples, Group G6 could significantly (*p* ≤ 0.05) decrease β-glucosidase, nitroreductase and azoreductase concentrations, while Group G5 significantly (*p* ≤ 0.05) reduced activities of only β-glucoronidase and nitroreductase as compared to DMH control group (G2).

### 2.5. Bacterial Populations

Fecal bacterial concentration data are shown in Table 4. Microbial concentrations of beneficial bacteria (bifidobacteria and lactobacilli) were increased in all prebiotics treatment groups and decreased concentrations of harmful bacteria were observed in these treatment groups. Among all prebiotic treatment groups, only Group G5 showed significantly (*p* ≤ 0.05) higher concentrations of lactobacilli as compared to Groups G2 (basal diet + DMH) and G1 (Basal Diet Control Group). Groups G5 and G6 had significantly (*p* ≤ 0.05) higher population of bifidobacteria as compared to Group G2. No significant difference was observed in concentration of enterococci among all groups; however, Group G6 had significantly (*p* ≤ 0.05) lower concentrations of clostridia as compared to Group G2.

## 3. Discussion

CRC is prevalent worldwide and annually 500,000 people die. Western developed countries have higher incidence of CRC [22]. Diet is considered a major factor as it can play either good or bad role for CRC development. Diet rich in sweets, alcohol, refined grains, red meat and processed meat increases CRC risk and diet rich in dairy products, fruits, vegetables, fish, olive oil and fiber is considered beneficial for cancer protection [23]. The current study was performed to assess protective role of prebiotics GalOS and inulin (as positive control) against various biomarkers of CRC. Prebiotics are bioactive ingredients of recent past and have several confirmed and postulated impacts on gastrointestinal microbiota and their metabolism especially in colon [24]. The prebiotics, probiotics [25] and synbiotics [26] have applied in various CRC induced rat models and have shown protective as well as preventive roles against tumor development and various other early biomarkers [17,27]. To date, studies to find protective roles of GalOS in CRC are very limited. The primary glycosidic linkage in GalOS used in previous studies related to cancer was β-1,4 and no product with β-1,6 and/or β-1,3 linkage have been used yet. Furthermore, purity of GalOS in these studies was 58.8% with other mono- and disaccharides [20,21]. To our best knowledge, prebiotic GalOS that contains β-1,6 and/or β-1,3 glycosidic linkage is used for the first time in the present study with purity of 30%. Our data demonstrate that high dose of GalOS provide a significant protective effect against development of colon cancer. It was revealed that the inhibition of ACF formation was significantly higher in inulin and high dose GalOS groups. The results in this study suggest that GalOS had dose-dependent resistance against body weight loss in DMH-induced groups and high dose groups showed higher levels of cecal and fecal SCFAs.

ACF are considered very good biomarkers for initiation stage of colon cancer and can be easily detected in colon mucosa of patients at high risk of CRC development [13]. No ACF formation was detected in Group G1 (control group fed on basal diet). Data of ACF incidence indicate that DMH successfully induced ACF in all DMH treated animals. Highest number of ACF was observed in Group G2 (basal diet + DMH) in all parts of colon. These results demonstrate that prebiotics have significant effect on reduction of DMH-induced ACF, particularly Groups G5 and G6. The reduction of ACF in rats in the prebiotics groups may be due to induction of apoptosis in colonic crypts, modulation of microbiota, increased enzymatic activity and improved concentrations of SCFAs, as all these factors are very much important for normal development and smooth functioning of colonic epithelial cells [17,28]. Because of prebiotic fermentation in colon, SCFAs are produced and it is believed that, among these SCFAs, butyrate plays an important role in inhibiting tumor cells [29,30]. These results of ACF inhibition support previous investigation by Verma and Shukla [18] in which they studied the effects of inulin and lactulose on different biomarkers of CRC in-vivo. They also concluded that prebiotic inulin significantly reduced ACF count as compared to only DMH treated animals. Findings of another study conducted by Wijnands, Schoterman, Bruijntjes, Hollanders and Woutersen [21] using GalOS as treatments support our findings of protective effect of GalOS on ACF inhibition.

Elevated levels of SCFAs in prebiotic groups of present study are in accordance with previous studies in which higher concentration of SCFAs were observed in rats after inulin supplementations [31,32,33]. Another study conducted by Wijnands, Schoterman, Bruijntjes, Hollanders and Woutersen [21] using commercial GalOS having β-1,4 linkage also showed higher levels of SCFAs in high dose GalOS groups which strengthen our findings. We also observed that groups which had higher levels of these SCFAs the total number of ACF count was low in those groups, it might be due to the increased levels of butyrate, which is considered important for tumor suppression as it plays selective roles in normal and colonic tumor cells. It induces apoptosis in cancerous cells and protects colon from genotoxic carcinogens [30,34]. Infusion of high dose of butyrate in distal colon has shown more than 60% reduction in the formation of tumors in azoxymethane induced rats as compared to control groups [35].

It is well established that microbial enzymes (β-glucuronidase, β-glucosidase, azoreductase and nitroreductase) can influence carcinogenesis development [18,36]; increased activity of β-glucuronidase is associated with increased incidence of neoplasms in colon [37]. One of the conditions for prebiotics is to escape the digestion in upper gastrointestinal tract so that beneficial microbiota (bifidobacteria and lactobacilli) in lower tract utilize undigested prebiotics in colon [38]. Decreased activity of these enzymes in prebiotics treatment groups in present study may be due to the increased population of health beneficial microbiota as previous studies have also documented the increased growth of probiotics and decreased growth of pathogens and low activity of pathogen bacterial enzymes after supplementation of prebiotics [26,28,33,38].

About 100 trillion microbial organisms are resident in adult human gut; these microbial organisms are collectively called microbiota [39]. Human health status directly and indirectly is dependent on resident microbiota, as these microbes ferment various food substances in colon that are left undigested and unabsorbed in small intestine. In gastrointestinal tract, carbohydrate and proteolytic are two main types of fermentations. SCFAs (butyrate, acetate and propionate) are major by-products of carbohydrate fermentation and are considered beneficial for health. However, end products of proteolytic fermentation (phenolic compounds, amines, ammonia, N-nitroso compounds and indoles) can be toxic to the host [40]. We found increased concentrations of beneficial bacteria (lactobacilli and bifidobacteria) in our study. As these microbes are known to ferment prebiotics in colon, we also observed elevated levels of SCFAs in prebiotic treatment groups. As increased populations of beneficial bacteria is supposed to suppress the concentrations of pathogens, we observed decrease in enterococci and clostridia populations as these microbes are efficient producers of β-glucoronidase and β-glucosidase [41], which might be one of the reasons we observed lower concentrations of these enzymes in our all prebiotic treatment groups. Interestingly, growth of lactobacilli was highest in Groups G4 and G5, which received prebiotics GalOS as compared to bifidobacterium, thus strengthening the previous findings by Tzortzis et al. that prebiotics results in maximum growth of those bacteria which have used as source of enzyme for their production [42,43]. In present study, *Lactobacillus reuteri* L103 was used as source of β-gal for GalOS production.

## 4. Materials and Methods

### 4.1. Chemicals and Reagents

The chemicals and reagents used in the study were of analytical grade, procured form Merck Chemicals (Darmstadt, Germany) unless stated. The prebiotic inulin was purchased from Cargill (Minneapolis, MN, USA). The *ortho*-nitrophenyl-β-galactopyranoside (*o*NPG), DMH, phenolphthalein-β-d-glucuronide, *m*-nitrobenzoic acid and nitrophenyl-β-d-glucoside were purchased from Sigma-Aldrich (St. Louis, MO, USA).

### 4.2. Animals, Treatments and Diets

Six-week-old male Wister rats (*n* = 90) were procured from Institute of Biochemistry and Biotechnology, UVAS, Lahore and were housed in stainless steel cages under standard conditions of temperature (23 ± 2 °C) and humidity (55 ± 10%) in a 12 h light–dark cycle. All experimental protocols used herein were approved by the “University Ethical Review Committee (DAS No.: 5372 Date: 14-04-2017) for the use of Laboratory Animals”. Rats were fed on standard diet for 1-week acclimatization period before beginning the experiment. After acclimatization period, 90 rats were equally and randomly divided into six groups. G1 was control group (CG) fed on AIN-93G/M as control basal diets [44]. Group G2 (basal diet + DMH) was DMH control group. Treatments with prebiotics GalOS were given to Groups G3–G5 while Group G6 was positive control and was given prebiotic inulin treatment. Prebiotics doses were calculated by using the human equivalent dose (HED) equation: HED (mg/kg) = animal dose in mg/kg × (animal weight in kg/human weight in kg)^0.33^ [45]. Groups G3, G4 and G5 received 76 mg (HED = 4 g), 114 mg (HED = 6 g) and 151 mg (HED = 8 g) GalOS, respectively, and Group G6 received 114 mg (HED = 6 g) of inulin.

DMH treatment (40 mg/kg body weight) was given to Groups G2–G6 through four subcutaneous injections prepared in 1 mM Ethylenediaminetetraacetic acid (EDTA) solution of pH 6.0, twice a week for 2 weeks [19,46]. The control group was treated with same concentrations of EDTA in a similar pattern. After completing the DMH doses, prebiotics treatments were started to respective groups for 16 weeks. Body weight of each rat in each group was recorded weekly and doses of prebiotics to respective groups were given according to mean body weight of each group. To make conditions similar to all groups, all prebiotics were given orally through tube feeding while control groups (G1 and G2) were administered same amount of water. After 16 weeks, all animals were killed using exsanguination under anesthesia with sodium pentobarbital (45 mg/kg body weight).

### 4.3. Preparation of GalOS

The β-galactosidase (β-gal) was produced from *Escherichia coli* BL21 containing gene from *L. reuteri* L103 following procedure explained by Iqbal et al. [47]. The enzyme activity was measured for chromogenic substrate *o*NPG and natural substrate lactose following methods by Splechtna et al. [48]. The crude β-gal was used for the production of GalOS through bioconversion of lactose by following method of Splechtna, Nguyen, Steinböck, Kulbe, Lorenz and Haltrich [48]. The transgalactosylated mixture were analyzed for glucose using GOPOD assay kit (K-GLUC) however galactose and lactose were measured using Lactose/Galactose assay kit (K-LACGAR) from Megazyme (Wicklow, Ireland) by following protocol given in the provided documents. The main glycosidic linkage in present prebiotic GalOS was β-1,6 with main products β-d-Galp-(1–6)-d-Glc, β- d-Galp-(1–6)-d-Gal and β-d-Galp-(1–6)-Lac, followed by β-1,3 linked products, i.e., β- d-Galp-(1–3)-d -Gal and β-d-Galp-(1–3)-Lac. While no product with β1–4 linkage was identified [47,48] as composition determined by High performance anion exchange chromatography with pulsed amperometric detection (HPAEC-PAD) and capillary electrophoresis.

### 4.4. Measurement of Body Mass

Changes in body weight and feed intake were measured on weekly basis using weighing scale (SCL66110 Olympia Plus Kent Scientific Corporation, Torrington, WY, USA).

### 4.5. Aberrant Crypt Foci (ACF) Analysis

ACF analysis were performed using a method previously described by Bird [49]. Briefly, after sacrificing animals, colon was removed starting from cecum to anus using sharp blade and colon was opened along longitudinal axis, remaining undigested material in colon was gently washed away by saline solution. Colon was fixed in 10% formalin at room temperature for 24 h. Methylene blue (0.2%) in saline was used for staining of colon. ACF were identified by microscopy at a magnification of ×40. Colon was divided into three parts distal (near rectum), middle and proximal colon (near cecum). The number of ACF in three parts of colon was added to get total number of ACF.

### 4.6. Samples Collection and Enzyme Analysis

The rectal region of rats was gently squeezed to get fresh fecal sample while cecum sample was collected during opening of large intestine after sacrificing each animal. Both types of samples were processed immediately after collection. Detailed procedures to measure activities of nitroreductase, β-glucuronidase and β-glucosidase in cecum and fecal samples are explained in our previous study [50] and azoreductase activity was determined following Goldin and Gorbach [36] method.

### 4.7. Short Chain Fatty Acids (SCFAs)

The amounts of SCFAs in cecal and fecal contents were determined using gas chromatography (Agilent 6890 Plus, Santa Clara, CA, USA) following method described by Zhao et al. [51].

### 4.8. Microbial Analysis

Bacterial groups from fecal samples were identified using plate count methods as previously described by Van de Wiele et al. [52]. Specific media (Oxoid, Hampshire, UK) were used for each bacterial group, including Rogosa agar (lactobacilli), raffinose Bifidobacterium agar (bifidobacteria), Enterococcus agar (enterococci), and tryptose sulfite cycloserin agar (clostridia).

### 4.9. Statistical Analysis

All collected data were expressed as mean ± standard error (SE). One-way analysis of variance in SPSS software (ver. 18, IBM SPSS Software Company, Chicago, IL, USA) was applied to analyze variation among groups. Duncan Multiple Range (DMR) test was applied for post-hoc analysis in significantly different results. Differences were considered significant at *p* < 0.05.

## 5. Conclusions

It can be concluded that novel prebiotic GalOS having β-1,6 and β-1,3 glycosidic linkage has dose dependent protective effects on various biomarkers of CRC and are more protective in higher doses. Further studies are required at gene expression level to understand the mechanisms of action of these prebiotics in the protection of CRC.

## Figures and Tables

**Table 1:** Effect of prebiotic treatments on aberrant crypt foci (ACF) in proximal, middle and distal colon in 1,2-dimethylhydrazine dihydrochloride (DMH)-initiated and non-initiated animals (*n* = 15).

ACF	G1 (Basal Diet Control Group)	G2 (DMH Control Group)	G3 (DMH + GalOS 76 mg)	G4 (DMH + GalOS 114 mg)	G5 (DMH + GalOS 151 mg)	G6 (DMH + Inulin 114 mg)
ACF/proximal colon	ND	27.4 ± 2.10 ^a^	24.7 ± 3.11 ^a,b^	18.7 ± 2.56 ^b,c^	14.3 ± 1.78 ^c^	16.4 ± 2.83 ^c^
ACF/middle colon	ND	44.7 ± 2.98 ^a^	42.3 ± 2.86 ^a,b^	34.3 ± 3.8 ^b,c^	22.9 ± 3.45 ^d^	28.3 ± 3.23 ^c,d^
ACF/distal colon	ND	119.1 ± 5.56 ^a^	115.3 ± 7.22 ^a,b^	110.4 ± 4.63 ^a,b^	101.7 ± 3.32 ^b,c^	90.3 ± 3.31 ^c^
Total ACF/colon	ND	191.2 ± 6.46 ^a^	182.4 ± 7.92 ^a^	163.4 ± 4.72 ^b^	139.0 ± 3.24 ^c^	135.1 ± 3.73 ^c^
Percent inhibition of total ACF	-	-	4.6	14.53	27.3	29.3

Values are expressed as Mean ± SE. Means in the same row with different superscript are significantly different (*p* ≤ 0.05). DMH, 1,2-dimethylhydrazine dihydrochloride (4 × 40 mg/kg b.wt., s.c.); ND, Not detected.

**Table 2:** Effect of prebiotic treatments on short chain fatty acids (SCFAs) concentrations of cecal and fecal contents in 1,2-dimethylhydrazine dihydrochloride (DMH) initiated and non-initiated animals (*n* = 15).

Parameters	G1 (Basal Diet Control Group)	G2 (DMH Control Group)	G3 (DMH + GalOS 76 mg)	G4 (DMH + GalOS 114 mg)	G5 (DMH + GalOS 151 mg)	G6 (DMH + Inulin 114 mg)
Cecum						
Acetate	80.8 ± 3.41 ^c^	79.2 ± 3.28 ^c^	82.8 ± 3.59 ^b,c^	86.2 ± 3.85 ^a,b,c^	92.4 ± 3.49 ^a,b^	94.9 ± 3.83 ^a^
Propionate	21.9 ± 2.03 ^a,b^	20.6 ± 1.85 ^b^	22.1 ± 2.11 ^a,b^	23.4 ± 1.78 ^a,b^	26.1 ± 1.54 ^a,b^	26.6 ± 1.44 ^a^
Butyrate	14.1 ± 1.11 ^c^	13.9 ± 1.03 ^c^	14.8 ± 0.93 ^b,c^	16.4 ± 1.21 ^a,b,c^	18.5 ± 1.36 ^a^	17.8 ± 1.09 ^a,b^
Fecal						
Acetate	51.7 ± 2.58 ^b^	50.6 ± 2.71 ^b^	53.2 ± 3.11 ^b^	56.8 ± 2.35 ^a,b^	62.1 ± 3.13 ^a^	64.8 ± 2.67 ^a^
Propionate	14.3 ± 0.99 ^c,d^	14.1 ± 0.79 ^d^	15.5 ± 0.68 ^c,d^	17.4 ± 1.05 ^b,c^	20.3 ± 1.26 ^a,b^	21.2 ± 1.34 ^a^
Butyrate	5.2 ± 0.51 ^b^	4.92 ± 0.48 ^b^	5.5 ± 0.53 ^b^	6.1 ± 0.53 ^a,b^	7.3 ± 0.57 ^a^	7.5 ± 0.67 ^a^

Values are expressed as Mean ± SE. Means in the same row with different superscript are significantly different (*p* ≤ 0.05). DMH, 1,2-dimethyl hydrazine dihydrochloride (4 × 40 mg/kg b.wt., s.c.); SCFAs, μmol/g.

**Table 3:** Effect of prebiotic treatments on cecal and fecal enzyme activities in 1,2-dimethylhydrazine dihydrochloride (DMH) initiated and non-initiated animals (*n* = 15).

Enzymes	G1 (Basal Diet Control Group)	G2 (DMH Control Group)	G3 (DMH + GalOS 76 mg)	G4 (DMH + GalOS 114 mg)	G5 (DMH + GalOS 151 mg)	G6 (DMH + Inulin 114 mg)
Cecum						
β-Glucosidase	0.82 ± 0.11 ^a,b^	0.96 ± 0.11 ^a^	0.94 ± 0.09 ^a^	0.80 ± 0.10 ^a,b^	0.77 ± 0.11 ^a,b^	0.59 ± 0.09 ^b^
β-Glucoronidase	3.43 ± 0.18 ^a,b^	3.52 ± 0.19 ^a^	3.25 ± 0.12 ^a,b^	3.12 ± 0.18 ^a,b^	2.96 ± 0.15 ^b^	2.99 ± 0.19 ^a,b^
Nitroreductase	4.38 ± 0.29 ^a^	4.47 ± 0.28 ^a^	4.27 ± 0.25 ^a^	3.83 ± 0.25 ^a,b^	3.44 ± 0.19 ^b^	3.26 ± 0.17 ^b^
Azoreductase	11.67 ± 1.01 ^a^	11.42 ± 0.56 ^a,b^	10.08 ± 0.67 ^a,b^	10.17 ± 0.81 ^a,b^	9.16 ± 0.79 ^b^	9.25 ± 0.72 ^a,b^
Fecal						
β-Glucosidase	0.72 ± 0.11 ^a,b^	0.91 ± 0.10 ^a^	0.76 ± 0.08 ^a,b^	0.69 ± 0.09 ^a,b^	0.64 ± 0.11 ^a,b^	0.53 ± 0.09 ^b^
β-Glucoronidase	2.97 ± 0.25 ^a^	3.07 ± 0.16 ^a^	2.93 ± 0.10 ^a,b^	2.72 ± 0.14 ^a,b^	2.43 ± 0.15 ^b^	2.55 ± 0.16 ^a,b^
Nitroreductase	3.13 ± 0.18 ^a^	3.18 ± 0.19 ^a^	2.86 ± 0.15 ^a,b^	2.76 ± 0.15 ^a,b^	2.49 ± 0.17 ^b^	2.55 ± 0.18 ^b^
Azoreductase	8.17 ± 0.51 ^a,b^	8.42 ± 0.42 ^a^	7.92 ± 0.47 ^a,b^	7.41 ± 0.59 ^a,b^	6.91 ± 0.45 ^a,b^	6.67 ± 0.57 ^b^

Values are expressed as Mean ± SE. Means in the same row with different superscript are significantly different (*p* ≤ 0.05). DMH, 1,2-dimethylhydrazine dihydrochloride (4 × 40 mg/kg b.wt., s.c.); β-Glucosidase, μg/min/mg cecal or fecal protein; β-Glucoronidase, μg/min/mg cecal or fecal protein; Nitroreductase and Azoreductase, μg/h/mg cecal or fecal protein.

**Table 4:** Effect of prebiotic treatments on fecal microbial concentrations in 1,2-dimethylhydrazine dihydrochloride (DMH) initiated and non-initiated animals (*n* = 15).

Microbes	G1 (Basal Diet Control Group)	G2 (DMH Control Group)	G3 (DMH + GalOS 76 mg)	G4 (DMH + GalOS 114 mg)	G5 (DMH + GalOS 151 mg)	G6 (DMH + Inulin 114 mg)
Lactobacillus	7.18 ± 0.24 ^b^	7.14 ± 0.17 ^b^	7.39 ± 0.18 ^b^	7.64 ± 0.09 ^a,b^	7.93 ± 0.16 ^a^	7.52 ± 0.18 ^a,b^
Bifidobacteria	8.11 ± 0.14 ^c,d^	8.07 ± 0.15 ^d^	8.18 ± 0.14 ^c,d^	8.24 ± 0.13 ^b,c,d^	8.51 ± 0.15 ^b,c^	9.12 ± 0.11 ^a^
Clostridia	8.82 ± 0.12 ^a,b^	8.92 ± 0.11 ^a^	8.77 ± 0.15 ^a,b^	8.59 ± 0.20 ^a,b,c^	8.51 ± 0.14 ^a,b,c^	8.45 ± 0.11 ^b,c^
Enterococci	7.42 ± 0.11	7.48 ± 0.10	7.53 ± 0.18	7.37 ± 0.17	7.22 ± 0.15	7.27 ± 0.09

Values are expressed as Mean ± SE. Means in the same row with different superscipts are significantly different (*p* ≤ 0.05). DMH, 1,2-dimethylhydrazine dihydrochloride (4 × 40 mg/kg b.wt., s.c.) log_10_ CFU/g wet weight of fecal.

## Abbreviations

CRC	Colorectal cancer
ACF	Aberrant crypt foci
GalOS	Galacto-oligosaccharides
DMH	1,2-Dimethylhydrazine dihydrochloride
SCFAs	Short chain fatty acids
ITF	Inulin-type fructans
DP	Degree of polymerization
FOS	Fructo-oligosaccharides
*o*NPG	*Ortho*-nitrophenyl-β-galactopyranoside
HED	Human equivalent dose
EDTA	Ethylenediaminetetraacetic acid
β-Gal	β-Galactosidase
HPAEC-PAD	High performance anion exchange chromatography with pulsed amperometric detection
